# FEN1 is critical for rapid single-strand break repair in G1 phase

**DOI:** 10.1093/nar/gkaf710

**Published:** 2025-07-22

**Authors:** Kamila Burdova, Richard Hailstone, Hana Hanzlikova, Keith W Caldecott

**Affiliations:** Laboratory of Genome Dynamics, Institute of Molecular Genetics of the Czech Academy of Sciences, 142 20 Prague 4, Czech Republic; Genome Damage and Stability Centre, University of Sussex, Falmer, Brighton BN1 9RQ, United Kingdom; Genome Damage and Stability Centre, University of Sussex, Falmer, Brighton BN1 9RQ, United Kingdom; Laboratory of Genome Dynamics, Institute of Molecular Genetics of the Czech Academy of Sciences, 142 20 Prague 4, Czech Republic; Institute of Animal Pathology, Vetsuisse Faculty, University of Bern, 3012 Bern, Switzerland; Genome Damage and Stability Centre, University of Sussex, Falmer, Brighton BN1 9RQ, United Kingdom

## Abstract

Flap endonuclease 1 (FEN1)-dependent long-patch repair has been considered a minor sub-pathway of DNA single-strand break repair (SSBR), activated only when short-patch repair is not feasible. However, the significance of long-patch repair in living cells remains unclear. Here, we employed human RPE-1 cells with FEN1 deletion to compare the requirements for short- and long-patch pathways for the rapid repair of various types of DNA single-strand breaks (SSBs). We found that SSBs arising from abortive topoisomerase 1 activity are repaired efficiently without FEN1. In contrast, the rapid repair of SSBs arising during base excision repair following treatment with methyl methanesulphonate (MMS) or following treatment with hydrogen peroxide (H_2_O_2_) exhibits an unexpectedly high dependence on FEN1. Indeed, in G1 phase, FEN1 deletion slows the rate of SSBR to a similar or even greater extent than deletion of the short-patch repair proteins XRCC1 or POLβ. As expected, the combined deletion of FEN1 with XRCC1 or POLβ has an additive or synergistic effect, severely attenuating SSBR rates after MMS or H_2_O_2_ exposure. These data highlight an unanticipated requirement for FEN1 in the rapid repair of SSBs in human cells, challenging the prevailing view that long-patch repair is a minor sub-pathway of SSBR.

## Graphical Abstract

**Graphical Abstract:**
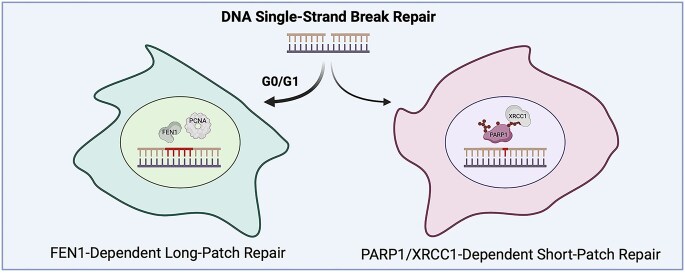

## Introduction

Single-strand breaks (SSBs) are among the most common DNA lesions arising in cells, and are induced both directly as a result of oxidative damage to deoxyribose and indirectly during the base excision repair (BER) of damaged DNA bases [1]. In addition, SSBs can arise as intermediates of enzymatic processes critical for DNA replication and gene transcription, such as the maturation of Okazaki fragments, the abortive activity of topoisomerases, and the demethylation of 5-methylcytosine during epigenetic reprogramming [1–3]. The rapid repair of SSBs is thus critical to maintain genomic integrity at a level compatible with cellular and organismal health. This is best illustrated by the existence of at least six hereditary genetic diseases in which one of four single-strand break repair (SSBR) genes are mutated [4].

Current models propose that SSBR is conducted by two distinct pathways, denoted short-patch repair and long-patch repair [4–6]. Short-patch repair involves the replacement of a single nucleotide at the damage site, typically carried out by DNA polymerase β (POLβ), whereas long-patch repair involves replacement of ∼2–15 nucleotides through the action of POLβ and/or DNA polymerase δ (POLδ) [7–14]. Short-patch repair is mediated by proteins that interact with the scaffold protein XRCC1, including aprataxin, polynucleotide kinase phosphatase (PNKP), POLβ, and DNA ligase III [7, 15–19]. In contrast, long-patch repair is facilitated by proteins that associate with proliferating cell nuclear antigen (PCNA), such as POLδ, flap endonuclease 1 (FEN1), and DNA ligase I [11–13, 20–23]. A defining feature of long-patch repair is the involvement of FEN1, which is responsible for removing the 5′-single-stranded flap generated during long-patch gap filling [13, 20].

Early biochemical studies found that short-patch repair is the major sub-pathway for BER, accounting for >70% of BER reactions [24–26]. However, later studies reported that long-patch BER occurs at a similar or greater frequency than short-patch repair [27, 28]. One factor that can affect the balance between these pathways is cell type. For example, muscle cells differentiated from human iPSC primarily employ short-patch BER during active cytosine demethylation, whereas in differentiated neurons, both short- and long-patch BER are commonly employed [29, 30]. Another factor affecting sub-pathway choice is the chemistry of the 5′-termini at SSBs [31]. For example, during BER, bifunctional DNA glycosylases create 5′-termini with canonical 5′-phosphates, which enable rapid ligation following single nucleotide gap filling, thereby favouring short-patch repair. In contrast, monofunctional DNA glycosylases create 5′-abasic sites that require excision by the deoxyribose phosphodiesterase (dRpase) activity of POLβ [32], which may increase the half-life of the SSB and the chance of longer repair patches. Indeed, reduced or oxidized 5′-abasic sites are resistant to dRpase activity and can only be repaired by long-patch repair [24, 32, 33]. Finally, the repair of ‘direct’ SSBs arising from hydrogen abstraction and disintegration of oxidized deoxyribose can be achieved by either short-patch or long-patch repair [14, 18].

Here, we employed human cells with deletions of FEN1 and/or XRCC1 or POLβ to examine the relative contributions of long-patch and short-patch repair, respectively, for the rapid repair of different types of SSBs in living cells. Our findings reveal that FEN1 has a significantly greater impact on SSB repair rates than previously anticipated, challenging the notion that long-patch repair is a minor sub-pathway.

## Materials and methods

### Cell lines, cell culture, chemicals, and antibodies

Human wild-type hTERT RPE-1 cells (denoted as RPE-1), U2OS cells, and FEN1-deleted derivatives (*FEN1^−/−^* RPE-1 clone #21/6; *FEN1^−/−^* U2OS clone #6; denoted as koFEN1) were described previously [34]. HEK293 Flp-In™ T-REx™ were obtained from Life Technologies. Cells were cultured grown in DMEM/F-12 or DMEM media (Gibco) supplemented with 10% serum and penicillin/streptomycin at 37°C, 5% CO_2_, and 3–5% oxygen. Cells were tested regularly for mycoplasma contamination. To synchronize RPE-1 cells into G0/G1 phase, they were grown to confluency and kept confluent in full media for 3 days followed by incubation in low-serum media for further 3 days. To induce DNA SSBs, cells were treated with 10–30 μM camptothecin (CPT) or 0.1 mg/ml methyl methanesulphonate (MMS) in complete media at 37°C for the times indicated, or with 100 μM hydrogen peroxide (H_2_O_2_) on ice for 10 min in serum-free media. CPT, MMS, and H_2_O_2_ were obtained from Sigma–Aldrich (C9911, 129925, and H1009, respectively). The PARP inhibitor olaparib (S1060; Selleck Chemicals) was used at a final concentration of 10 μM. Primary and secondary antibodies are indicated in Supplementary Table S1.

Gene editing of hTERT RPE-1 cells was performed using CRISPR/Cas9 plasmid as previously described [34–36]. For POLβ knockout cells (*POLβ^−/−^* RPE-1 clone #9; denoted as koPOLβ), gRNA sequences were GGCCGCCATGAGCAAACGGA and GCAGCGGGTCGTCTTCCGTG and clonal isolates were selected by cell sorting and knockout verified by western blot. FEN1/POLβ double-knockout cells (*FEN1^−/−^*/*POLβ^−/−^* RPE-1 clone #2; denoted as koFEN1/POLβ) were created in the *FEN1^−/−^* RPE-1 cell line described above.

To create XRCC1 knockout RPE-1 cells (*XRCC1^−/−^* RPE-1 clone #23; denoted as koXRCC1), synthetic gRNAs (GGUAGAGUAUGGGGUCCGAG and CAGACACUUACCGAAAAUGG) and recombinant Cas9 protein were used. Clonal isolates were obtained by dilution and knockout verified by western blotting. FEN1/XRCC1 double-knockout cells (*FEN1^−/−^*/*XRCC1^−/−^* RPE-1 clone #23; denoted as koFEN1/XRCC1) were created in the *FEN1^−/−^* RPE-1 cell line described above. Clones obtained by dilution were genotyped for insertion using PCR and verified by western blotting. To generate FEN1 knock-out in HEK293 FlipIN-TRex cells (*FEN1^−/−^* HEK293 #clone23; denoted as koFEN1), synthetic gRNA (UGUGGCCCCCAGUGCCAUCC) was used with Cas9 protein [34]. Clones were isolated by dilution and knockout verified by western blotting.

### Purification of FEN1 and proteofection

His-tagged wild-type and catalytically inactive FEN1 (R100A) were expressed in *Escherichia coli*BL21 (DE3) cells by induction with 0.1 mM isopropyl β-D-1-thiogalactopyranoside (IPTG) at 16°C overnight, as previously described [37, 38]. Bacterial cell pellets were resuspended in lysis buffer (20 mM Tris–HCl, pH 7.5, 1 M NaCl, 10 mM imidazole), sonicated, and centrifuged at 40 000 × *g* for 30 min at 4°C. Supernatant was filtered and loaded to 5-ml Ni HiTrap column. Column was extensively washed with lysis buffer and lysis buffer containing 40 mM imidazole. Protein was eluted using elution buffer (500 mM imidazole, 0.5 M NaCl, 20 mM Tris–HCl, pH 7.5), diluted with dH_2_O containing 1 mM dithiothreitol (DTT), and loaded to 5-ml HiTrap Q Sepharose. Flow-through containing FEN1 protein was collected, aliquoted, and stored at −80°C before use.

For complementation of cells by protein transfection (proteofection), cells were trypsinized, counted, washed with PBS, and resuspended in buffer R (Neon kit). Aliquot of 100 000 cells in 10 μl was mixed with 1 μg of purified FEN1 protein and electroporated by Neon 10-μl electroporation tip (1350 V/20 ms/2 pulses). Cells were left to recover for 15 min in full media before comet assay.

### Indirect immunofluorescence microscopy and sSTRIDE

Cells were seeded onto coverslips 1 day prior to treatment. After treatment, cells were washed with cold phosphate buffered saline (PBS) and pre-extracted on ice for 5 min using pre-extraction buffer (25 mM HEPES, pH 7.4, 50 mM NaCl, 1 mM EDTA, 3 mM MgCl_2_, 0.3 M sucrose, 0.5% Triton X-100). Cells were then fixed with 4% formaldehyde on ice, permeabilized with cold methanol/acetone for 5 min, and blocked with 5% BSA in PBS.

For sSTRIDE [39], cover slips were incubated with DNA Polymerase I (NEB) in buffer 2 (NEB) containing 5 μM nucleotides (biotin-7-dATP, biotin-16-dCTP, biotin-16-dUTP, and dGTP; Jena Bioscience) for 15 min at 37°C in a humified chamber. Reactions were stopped by 0.1 M EDTA and coverslips were washed with PBS.

Primary antibodies were applied in BSA for 1–2 h at room temperature (RT). Coverslips were washed three times with PBS, incubated with secondary antibodies in BSA for 30–60 min at RT, and washed three times with PBS. Nuclei were stained by incubation with DAPI in PBS for 5 min. Coverslips were then washed with dH_2_O, air-dried, and mounted using Vectashield. Images were acquired with an Olympus IX81 microscope equipped with the ScanR high-content module and a UPLXAPO 20×/0.8 or UPLXAPO 40×/0.95 DRY objective. The DAPI signal was used for intensity-based nuclear segmentation, and total fluorescence intensity in various channels was quantified. Signal intensities for different cell populations were analysed using FlowJo software (TreeStar).

### Alkaline comet assay

Cells were trypsinized and treated with the indicated genotoxins as described. Following treatment, cells were washed with ice-cold PBS, resuspended in ice-cold PBS and spread onto agarose-coated slides by mixing with a low-melting agarose (42°C). Cells were lysed in a lysis buffer (2.5 M NaCl, 100 mM EDTA, 10 mM Tris, pH 10, supplemented with 1% DMSO and 1% Triton X-100) for 1 h at 4°C. DNA was then unwound in alkaline running buffer (50 mM NaOH, 1 mM EDTA, 1% DMSO, pH >13) for 45 min. Slides were placed in a comet electrophoresis tank (Thermo) at 15 V for 25 min in a cold room. After electrophoresis, slides were neutralized using 0.4 M Tris (pH 7.4) for either 15 min (protocol II) or overnight (protocol I), followed by staining with SYBR Green (Sigma–Aldrich) diluted in PBS. For protocol I, images were scored immediately after staining using 20× objective and Comet Assay IV software (Perceptive Instruments). For protocol II, slides were washed three times with dH_2_O, air-dried, and images were captured using an Olympus IX81 microscope equipped with the ScanR high-content module and a UPLSAPO 10×/0.4 objective. Comets were scored from at least 50 cells per sample using CometScore 2.0 software.

### Cell fractionation

Cells were trypsinized and treated with the indicated genotoxins as described. After treatment, cells were washed with ice-cold PBS, resuspended in extraction buffer (25 mM HEPES, pH 7.4, 150 mM NaCl, 1 mM EDTA, 3 mM MgCl_2_, 0.3 M sucrose, 0.5% Triton X-100) supplemented with protease inhibitors (Roche), and incubated on ice for 15 min. Following incubation, samples were centrifuged at 20 000 × *g* for 15 min at 4°C. The chromatin pellet was washed three times with extraction buffer, resuspended in Laemmli sample buffer (2% SDS, 10% glycerol, 50 mM Tris–HCl, pH 6.8), boiled, and sonicated for subsequent analysis.

### Clonogenic cell survival assay

Indicated wild-type and single/double-knockout RPE-1 were synchronized into G0/G1 phase and seeded at 300 cells/10-cm plate in full media. After 4 h, cells treated with indicated doses of MMS for 30 min at RT. Plates were twice washed with PBS and incubated in drug-free media for 11 days. Cells were fixed with ethanol, stained with crystal violet, and colonies were counted. Relative surviving fraction of colonies was determined and plotted.

### SDS–PAGE and western blotting

To prepare whole-cell extracts, cells were treated with the indicated genotoxins as described, washed twice with ice-cold PBS, lysed in Laemmli sample buffer (2% SDS, 10% glycerol, 50 mM Tris–HCl, pH 6.8), boiled, and sonicated. Protein samples were resolved using Bis-Tris SDS–PAGE (sodium dodecyl sulphate–polyacrylamide gel electrophoresis) gels and transferred onto nitrocellulose membranes. Membranes were blocked with milk in PBS-T and incubated with primary antibodies overnight at 4°C. After washing with PBS-T, membranes were incubated with corresponding HRP-conjugated secondary antibodies, washed again, and developed (Optimax, Protec) using an ECL substrate (GE) and light-sensitive films (AGFA).

### Statistical analysis

All experiments comprised at least three independent biological replicates and statistical analysis employing ANOVA with Sidak or Holm/Sidak*post hoc* analysis using GraphPad Prism (GraphPad Software). Asterisks were used to indicate statistical significance (*P < .0332, **P < .0021, ***P < .0002, and ****P < .0001). All data are plotted as mean ± SD and only relevant statistically significant comparisons are shown.

## Results

### FEN1 facilitates efficient DNA single-strand break repair in human cells

**Figure 1.**
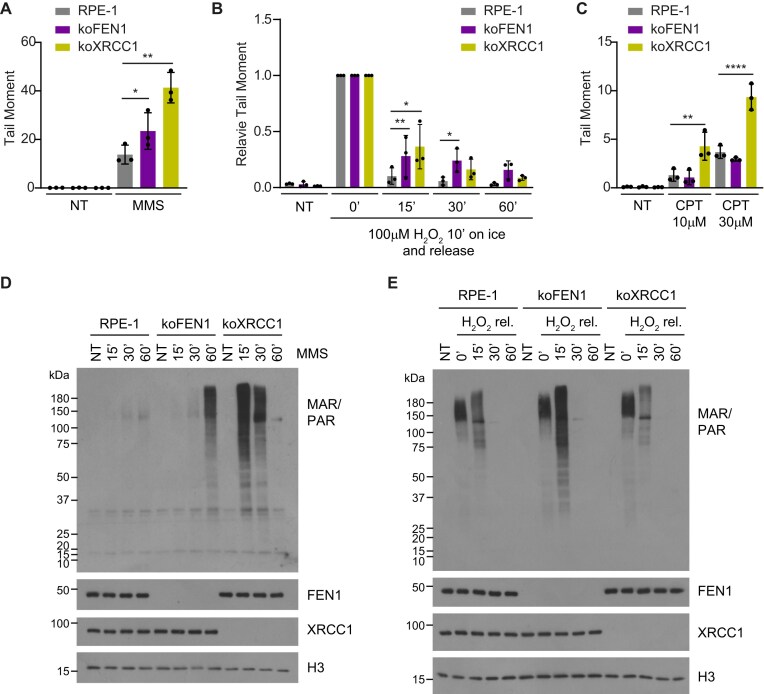
FEN1 facilitates efficient DNA SSBR in human cells. (**A**) DNA strand breaks in asynchronous wild-type (RPE-1), FEN1-deleted (koFEN1), and XRCC1-deleted (koFEN1) RPE-1 cells, measured by alkaline comet assays in untreated cells (NT) or after treatment with 0.1 mg/ml MMS for 15 min at 37°C. (**B**) DNA strand breaks in asynchronous wild-type (RPE-1), FEN1-deleted (koFEN1), and XRCC1-deleted (koXRCC1) RPE-1 cells, measured by alkaline comet assays in untreated cells (NT) or after treatment with 100 μM hydrogen peroxide (H_2_O_2_) on ice for 10 min, followed by release to drug-free media at 37°C for the indicated times. Tail moments are shown relative to values immediately after H_2_O_2_ treatment (absolute tail moments are provided in Supplementary Fig. S1D). (**C**) DNA strand breaks in asynchronous wild-type (RPE-1), FEN1-deleted (koFEN1), and XRCC1-deleted (koXRCC1) RPE-1 cells, measured by alkaline comet assays in untreated cells (NT) or after treatment with 10 or 30 μM CPT for 45 min at 37°C. (**D**) Levels of mono- and poly-ADP-ribosylated proteins (MAR/PAR) in wild-type (RPE-1), FEN1-deleted (koFEN1), and XRCC1-deleted (koXRCC1) RPE-1 cells in untreated conditions (NT) or after treatment with 0.1 mg/ml MMS at 37°C for the indicated times. Levels of FEN1, XRCC1, and histone H3 are shown as controls. (**E**) Levels of mono- and poly-ADP-ribosylated proteins (MAR/PAR) in wild-type (RPE-1), FEN1-deleted (koFEN1), and XRCC1-deleted (koXRCC1) RPE-1 cells in untreated conditions (NT) or after treatment with H_2_O_2_ as described in panel (B).

To examine the requirement for FEN1 for DNA SSBR in human cells, we conducted alkaline comet assays using FEN1-deleted cells (‘koFEN1’) generated via CRISPR/Cas9 editing, following treatment with various genotoxin agents that induce different types of physiologically relevant DNA SSBs. First, we employed MMS, which induces alkylated DNA bases that are converted into SSBs by AP endonuclease (APE1) during BER. FEN1 deletion significantly increased the accumulation of SSBs in asynchronous RPE-1 cell populations during MMS treatment, albeit to a lesser extent than XRCC1 deletion (‘koXRCC1’) (Fig. 1A). Importantly, this phenotype was rescued by protein transfection with wild-type FEN1, but not with catalytically dead FEN1 (CD) harbouring the nuclease active site mutation, R100A [37, 38] (Supplementary Fig. S1A). A similar defect in SSBR following MMS treatment was also observed in other human FEN1-deleted cell lines, including U2OS [34] and HEK293 (Supplementary Fig. S1B and C). Similar results were obtained following treatment with hydrogen peroxide (H_2_O_2_), which induces SSBs both directly by deoxyribose oxidation and fragmentation and indirectly as intermediates of BER. Indeed, surprisingly, the rate of SSBR in FEN1-deleted cells was as least as slow as that observed in XRCC1-deleted cells, following H_2_O_2_ (Fig. 1B and Supplementary Fig. S1D). In contrast to MMS and H_2_O_2_, FEN1-deleted cells did not accumulate higher levels of SSBs following treatment with CPT, which induces SSBs as a result of abortive topoisomerase 1 (TOP1) activity (Fig. 1C).

Further evidence supporting a greater than anticipated requirement for FEN1 in SSBR was obtained by measuring steady-state levels of ADP-ribosylation, which is an indirect indicator of SSBs. Consistent with the alkaline comet assays, these experiments demonstrated an increased accumulation and/or persistence of ADP-ribosylated proteins in FEN1-deleted and XRCC1-deleted RPE-1 cells, following treatment with MMS (Fig. 1D). Notably, the accumulation of ADP-ribose during MMS treatment occurred much faster in XRCC1-deleted cells than in FEN1-deleted cells (Fig. 1D), consistent with long-patch BER occurring at later times after MMS treatment than short-patch BER. The rapid decline in ADP-ribosylation in XRCC1-deleted cells at later times during MMS treatment has been reported previously, and reflects the exhaustion of NAD^+^ as a result of the greatly elevated activity of PARP1 [40]. Following H_2_O_2_ treatment, deletion of FEN1 resulted in a measurable impact on the rate at which ADP-ribosylation declined (Fig. 1E). This was not observed in XRCC1-deleted cells, however, despite both FEN1- and XRCC1-deficient cells displaying similarly delayed repair in alkaline comet assays (see above). This may reflect a limitation of using whole-cell extracts in western blotting to measure ADP-ribosylation, following H_2_O_2_, because we did detect a reduced rate of decline of H_2_O_2_-induced ADP-ribosylation in chromatin in XRCC1-deficient cells followed by indirect immunofluorescence (see Supplementary Fig. S3D). In agreement with the alkaline comet assays (Fig. 1C), FEN1 deletion did not elevate the level of ADP-ribosylation during treatment with CPT (Supplementary Fig. S1E).

To independently verify that FEN1-deleted cells exhibit reduced SSB repair following treatment with MMS and H_2_O_2_, we performed sSTRIDE [39], a highly sensitive, fluorescence microscopy-based technique that directly labels SSBs by 3′-end labelling. In agreement with our alkaline comet assays, sSTRIDE revealed a significantly greater accumulation of SSBs in FEN1-deleted cells during treatment with MMS, and a reduced rate of decline of SSBs following H_2_O_2_ treatment (Supplementary Fig. S1F and G). Similar results were observed in XRCC1-deleted cells following MMS treatment. Interestingly, sSTRIDE was unable to detect elevated H_2_O_2_-induced SSBs in XRCC1-deleted cells, presumably because these cells possess reduced PNKP 3′-phosphatase activity [18], which is required to convert H_2_O_2_-induced 3′-phosphate termini into 3′-hydroxyl termini that can support end labelling [39].

### FEN1 is required for rapid rates of SSBR in G1 phase of the cell cycle

**Figure 2.**
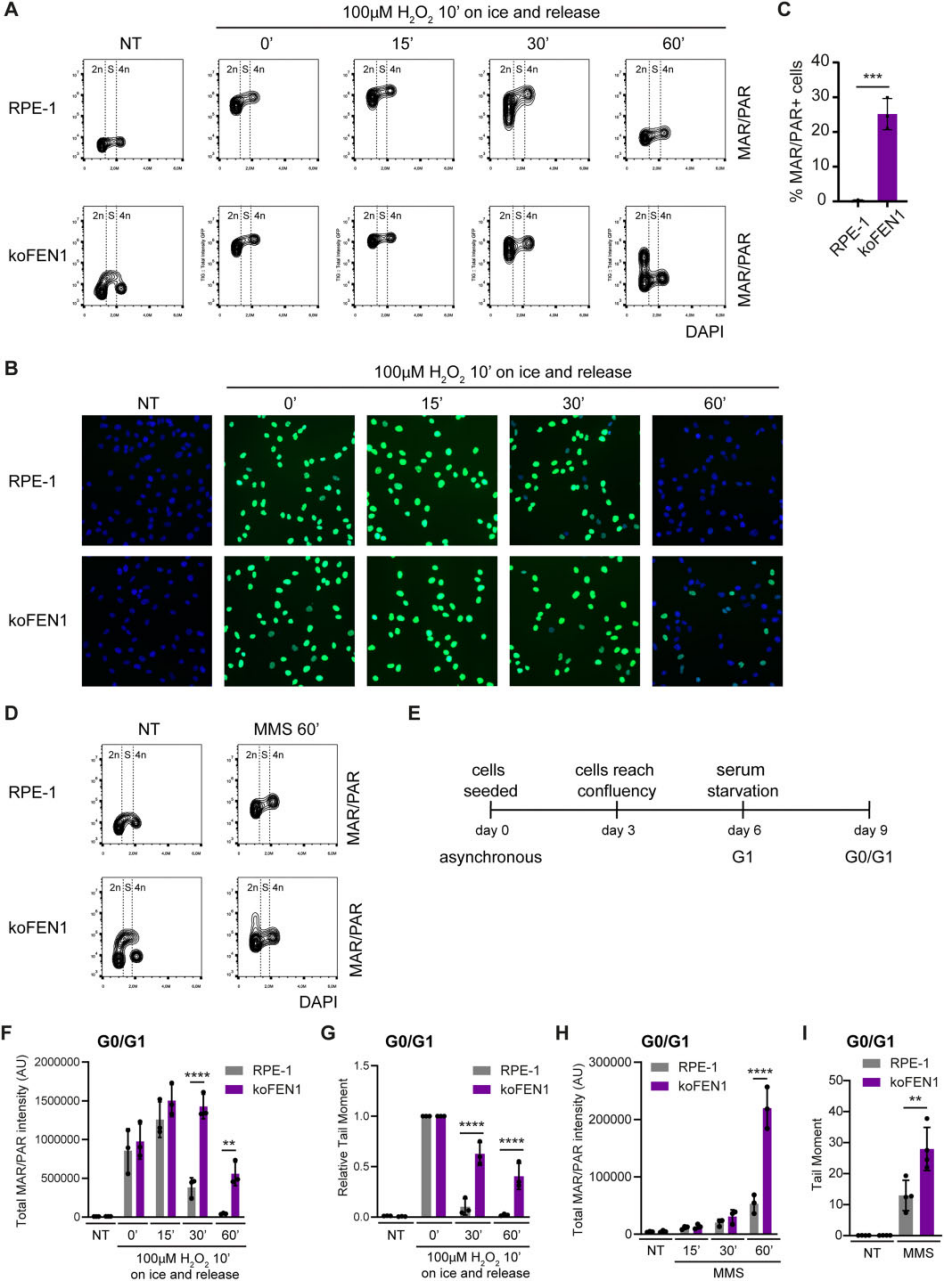
FEN1 is required for rapid rates of SSBR in the G1 phase. (**A**) Detection of chromatin-associated ADP-ribosylation (MAR/PAR) by indirect immunofluorescence in asynchronous wild-type (RPE-1) and FEN1-deleted (koFEN1) RPE-1 cells. Cells were left untreated (NT) or treated with 100 μM H_2_O_2_ on ice for 10 min, followed by incubation in drug-free media at 37°C for the indicated times. Contour plots show ADP-ribosylation levels (MAR/PAR) versus DNA content (DAPI). (**B**) Representative images of asynchronous cells shown in panel (A). Chromatin-bound nuclear mono- and poly-ADP-ribosylated proteins (MAR/PAR), visualized after detergent extraction, are show in green; nuclei are stained with DAPI (blue). (**C**) Quantification of MAR/PAR-positive 2*n* (G1 phase) cells in samples treated with 100 μM H_2_O_2_ on ice for 10 min, followed by incubation in drug-free media at 37°C for 60 min, as shown in panel (A). (**D**) Detection of chromatin-associated ADP-ribosylation (MAR/PAR) by indirect immunofluorescence in asynchronous cells, untreated (NT) or treated with 0.1 mg/ml MMS for 60 min. Contour plots of ADP-ribosylation versus DNA content (DAPI) are shown. (**E**) Schematic representation of the G0/G1 synchronization protocol. (**F**) Quantification of chromatin-associated ADP-ribosylation (MAR/PAR) by indirect immunofluorescence in G0/G1-synchronized cells, untreated (NT) or treated with 100 μM H_2_O_2_ on ice for 10 min, followed by incubation in drug-free media at 37°C for the indicated times. (**G**) DNA strand breaks in G1 phase wild-type (RPE-1), FEN1-deleted (koFEN1), and XRCC1-deleted (koXRCC1) RPE-1 cells, measured by alkaline comet assays after treatment or not (NT) with 100 μM H_2_O_2_ on ice for 10 min, followed by release into drug-free media at 37°C for the indicated times. Tail moments are presented relative to values immediately after H_2_O_2_ treatment (absolute tail moments are shown in Supplementary Fig. S2E). (**H**) Quantification of chromatin-associated ADP-ribosylation (MAR/PAR) by indirect immunofluorescence in G0/G1-synchronized cells, untreated (NT) or treated with 0.1 mg/ml MMS for the indicated times. (**I**) DNA strand breaks in G1 phase wild-type (RPE-1) and FEN1-deleted (koFEN1) RPE-1 cells, measured by alkaline comet assays in untreated cells (NT) or after treatment with 0.1 mg/ml MMS for 15 min at 37°C.

We next investigated whether the strong requirement of FEN1 for rapid SSBR following DNA methylation or DNA oxidation reflected its activity during S phase, in which FEN1 and other long-patch repair proteins play essential roles in DNA replication. To test this, we first examined ADP-ribosylation levels across the cell cycle in wild-type and FEN1-deleted RPE-1 cells using immunofluorescence. In asynchronous populations of untreated cells, FEN1-deleted cells exhibited higher levels of ADP-ribosylation in S phase compared to wild-type cells, consistent with the elevated unligated Okazaki fragments in these cells (Fig. 2A, panels labelled ‘NT’) [34, 41]. Following H_2_O_2_ treatment, ADP-ribosylation increased in all cell cycle phases in both wild-type and FEN1-deleted cells (Fig. 2A and B). Surprisingly, however, 60 min after H_2_O_2_ treatment, ADP-ribosylation was markedly higher in FEN1-deleted cells than in wild-type cells, in G1 phase (Fig. 2A–C and Supplementary Fig. S2A, left panel). Similar results were observed in FEN1-deleted cells in G1 phase following treatment with MMS (Fig. 2D and Supplementary Fig. S2B, left panel). While average ADP-ribosylation levels were also higher in FEN1-deleted S phase cells than in wild-type S phase cells following MMS- and H_2_O_2_ treatment, this difference was not statistically significant, and likely was influenced by unligated Okazaki fragments (Supplementary Fig. S2A and B, right panels). To better distinguish G1 cells, we performed MCM2 immunostaining in addition to DAPI staining. These experiments confirmed that the impact of FEN1 deletion on ADP-ribosylation was most pronounced in MCM2-negative (early G1 phase) cells than in MCM2-positive (late G1/early S phase) cells, 60 min after H_2_O_2_ treatment (Supplementary Fig. S2C).

To further verify the importance of FEN1 in G1 phase, we synchronized RPE-1 cells in G0/G1 by inducing growth arrest through confluency and serum starvation (Fig. 2E and Supplementary Fig. S2D). Consistent with the experiments described above, G0/G1-arrested FEN1-deleted cells exhibited significantly higher levels and/or prolonged persistence of ADP-ribosylation compared to wild-type cells following treatment with either H_2_O_2_ or MMS (Fig. 2F and H). Additionally, a pronounced defect was observed in G0/G1-arrested FEN1-deleted cells following H_2_O_2_ or MMS treatment when SSBR was measured directly using alkaline comet assays (Fig. 2G and I, Supplementary Fig. S2E and F). These findings demonstrate that FEN1 is essential for rapid rates of SSBR, particularly in the G1 phase of the cell cycle.

### FEN1-dependent repair is the dominant pathway for rapid SSBR in G1 phase

**Figure 3.**
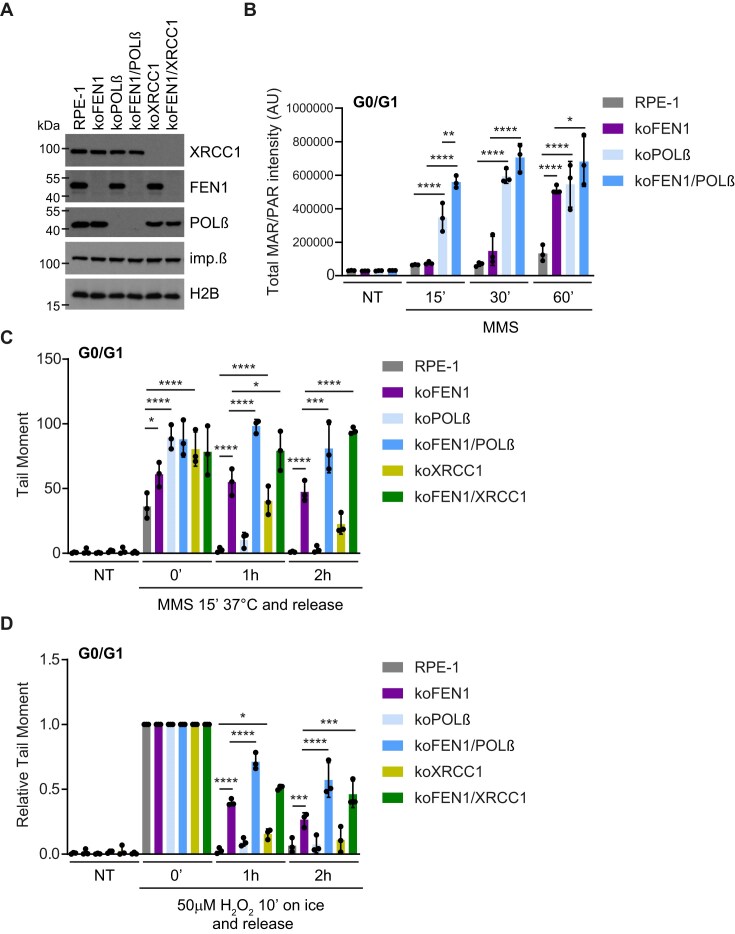
FEN1-dependent repair is the dominant pathway for rapid SSBR in G1 phase. (**A**) Levels of FEN1, XRCC1, and POLβ in whole-cell extracts from the indicated wild-type (RPE-1) and single/double-knockout RPE-1 cells, detected by western blotting. Levels of histone H2B and importin β were used as loading controls. (**B**) Quantification of chromatin-associated ADP-ribosylation (MAR/PAR) by indirect immunofluorescence in G0/G1-synchronized wild-type (RPE-1), FEN1-deleted (koFEN1), POLβ-deleted (koPOLβ), and FEN1/POLβ-deleted (koFEN1/POLβ) RPE-1 cells treated with 0.1 mg/ml MMS for the indicated times. (**C**) DNA strand breaks in G1-phase wild-type (RPE-1) and indicated single/double-knockout RPE-1 cell lines, measured by alkaline comet assays in untreated cells (NT) or after treatment with 0.1 mg/ml MMS for 15 min at 37°C, followed by incubation in drug-free media for the indicated times. Absolute tail moments are shown (relative tail moments are presented in Supplementary Fig. S3A). (**D**) DNA strand breaks in G1-phase wild-type (RPE-1) and the indicated single/double-knockout RPE-1 cell lines, measured by alkaline comet assays in untreated cells (NT) or after treatment with 50 μM H_2_O_2_ on ice for 10 min, followed by incubation in drug-free media at 37°C for the indicated times. Tail moments are presented relative to the values immediately after H_2_O_2_ treatment (absolute tail moments are shown in Supplementary Fig. S3C).

To examine the relative importance of short-patch and long-patch repair in G1 phase, we used cells lacking FEN1 and/or the short-patch repair protein POLβ or XRCC1 (Fig. 3A). Levels of ADP-ribosylation accumulated more quickly in POLβ-deleted cells (‘koPOLβ’) than in FEN1-deleted cells during treatment with MMS in G1 phase, consistent with the notion that short-patch repair is initiated earlier than long-patch repair (Fig. 3B). Deletion of either FEN1 or POLβ also resulted in the accumulation of DNA breaks induced during a 15-min MMS treatment in G1 phase, as measured by alkaline comet assays, with POLβ deletion having a greater impact than FEN1 depletion (Fig. 3C, ‘0’ time point). Surprisingly, however, MMS-induced breaks declined to background levels within 1 h in POLβ-deleted cells during a subsequent incubation in drug-free media, but did not significantly decline in FEN1-deleted cells (Fig. 3C and Supplementary Fig. S3A). In G1 cells lacking both POLβ and FEN1, MMS-induced SSBs persisted at even higher level than in cells lacking only FEN1, highlighting the additive or synergistic impact of inhibiting both short-patch and long-patch BER (Fig. 3C and Supplementary Fig. S3A). These results suggest that while POLβ-dependent short-patch BER is required during the 15-min exposure to MMS, it becomes redundant with long-patch repair over longer repair periods. Consistent with this, POLβ deletion alone did not invoke significant sensitivity to MMS in RPE-1 cells, whereas deletion of FEN1 did (Supplementary Fig. S3B). FEN1 deletion also slowed the repair of MMS-induced SSBs in G1 phase more than did XRCC1 deletion, and the deletion of both proteins again had an additive or synergistic impact (Fig. 3C and Supplementary Fig. S3A).

FEN1 deletion also had a much larger impact than did deletion of either XRCC1 or POLβ on the repair of H_2_O_2_-induced SSBs in G1 phase, and once again the deletion of FEN1 and either POLβ or XRCC1 had an additive or synergistic impact (Fig. 3D and Supplementary Fig. S3C). Similar results were observed when detecting SSBs indirectly by measuring levels of H_2_O_2_-induced ADP-ribosylation (Supplementary Fig. S3D, panel labelled ‘G1’). In contrast, and consistent with our earlier experiments, FEN1 deletion had a smaller impact on the persistence of H_2_O_2_-induced ADP-ribosylation in cells in S or G2 phase (Supplementary Fig. S3D, panels labelled ‘S’ and ‘G2’).

**Figure 4.**
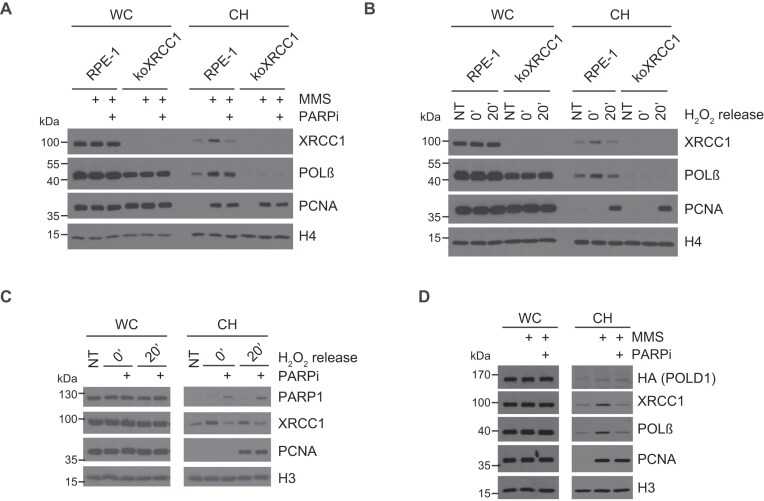
Recruitment of short-patch and long-patch repair proteins during SSB repair. (**A**) Western blot analysis of proteins in whole-cell lysate or in the detergent-extracted fraction of whole-cell extract (chromatin fraction; CH) from G0/G1-synchronized wild-type (RPE-1) and XRCC1-deleted (koXRCC1) RPE-1 cells after treatment with 0.1 mg/ml MMS for 1 h at 37°C. Where indicated, cells were pre-treated with 10 μM PARP inhibitor (PARPi; olaparib) for 15 min before MMS treatment. (**B**) Western blot analysis of protein extracts as above, after treatment of the indicated RPE-1 cells with 100 μM H_2_O_2_ in serum-free media on ice for 10 min, followed by release into fresh full media at 37°C for 20 min. (**C**) Western blot analysis of protein extracts after treatment of the indicated RPE-1 cells with 100 μM H_2_O_2_ as above. Where indicated, cells were pre-treated with 10 μM PARP inhibitor (PARPi; olaparib) for 15 min before H_2_O_2_ treatment. (**D**) Western blot analysis of protein extracts as above, following treatment of RPE-1 cells expressing HA-tagged POLD1 with 0.1 mg/ml MMS for 1 h at 37°C. Where indicated, cells were pre-treated with 10 μM PARP inhibitor (PARPi; olaparib) for 15 min before MMS treatment.

Finally, to confirm that short-patch and long-patch repair behaved as expected in our experiments, we compared the impact of PARP inhibition and XRCC1 deletion on the recruitment of short-patch and long-patch repair proteins into chromatin. As expected, PARP inhibition impeded the recruitment of XRCC1 and POLβ into chromatin following MMS or H_2_O_2_ treatment, consistent with their roles in short-patch repair (Fig. 4A, C, and D). Similarly, XRCC1 deletion prevented POLβ recruitment (Fig. 4A and B). In contrast, the recruitment of PCNA and POLD1, which function with FEN1 during long-patch BER, was unaffected by PARP inhibition or XRCC1 deletion (Fig. 4A–D). These data confirm the distinct roles of XRCC1–POLβ and FEN1–PCNA–POLD1 in short-patch and long-patch repair, respectively, in human cells.

## Discussion

Long-patch repair has been considered to be a relatively minor sub-pathway of SSBR, occurring, for example, only if short-patch repair cannot operate [24–26]. However, later studies have suggested that long-patch repair may occur at least as frequently as short-patch repair, during BER [27, 28]. In agreement with the latterstudies, we found that the loss of the long-patch repair protein FEN1 had a surprisingly large impact on SSBR rates following treatment with either MMS or H_2_O_2_. Indeed, in G1-arrested cells, the impact of FEN1 deletion was similar to or greater than that of deleting either XRCC1 or POLβ. For example, while loss of XRCC1, POLβ, or FEN1 each resulted in elevated SSB accumulation during a short (15-min) MMS treatment, the persistence of SSBs during a subsequent 2 h recovery in drug-free media was greatest in FEN1-deficient cells. Indeed, in POLβ-deficient cells, MMS-induced SSBs returned to background levels within 1 h. This residual repair was FEN1-dependent, because it was abolished by FEN1 deletion. MMS-induced SSBs also declined in XRCC1-deficient cells, albeit more slowly than in POLβ-deleted cells. The greater impact of XRCC1 loss during BER likely reflects its role in suppressing excessive PARP1 engagement and endogenous ‘trapping’ on BER intermediates, which slows short-patch BER [36, 40].

While it is not surprising that FEN1-dependent BER can compensate for loss of POLβ, our finding that FEN1 is essential for rapid rates of BER in wild-type cells was unexpected. Current models propose that FEN1-dependent long-patch BER is only required when POLβ is unable to facilitate short-patch BER, such as if the deoxyribose moiety at the 5′-terminus of the SSB is oxidized and cannot be removed by POLβ lyase activity [24, 32, 33]. However, MMS primarily generates abasic sites that are processed by APE1, producing a canonical substrate for POLβ-dependent short-patch BER. Nonetheless, based on the comparative levels of MMS-induced SSBs that accumulated and persisted in POLβ-deleted, FEN1-deleted, and FEN1/POLβ-deleted cells (see Fig. 3C), our data suggest that long-patch BER is required for more than half of BER events following DNA alkylation. One possible explanation is that POLβ ‘accidentally’ inserts an extra nucleotide during BER far more frequently than previously thought, thereby triggering the necessity for long-patch BER.

Whereas both MMS and H_2_O_2_ induce SSBs indirectly, as intermediates of BER, H_2_O_2_ additionally induces SSBs directly via the oxidation and disintegration of deoxyribose [42]. Similar to our findings in cells treated with MMS, in G1 phase, FEN1 deletion had a significantly greater impact on the rate of repair of H_2_O_2_-induced SSBs than did deletion of either XRCC1 or POLβ. Indeed, while POLβ was partially redundant for rapid repair of SSBs following treatment with MMS, it was completely dispensable following H_2_O_2_ treatment. XRCC1, however, remained more critical than POLβ, likely due to its role in recruiting and stimulating PNKP, the activity of which repairs the 3′-phosphate termini present at a large fraction (>50%) of ‘direct’ DNA breaks induced by H_2_O_2_ [18, 43–45]). The partial redundancy of POLβ and XRCC1 following H_2_O_2_ treatment was again largely the result of FEN1-dependent repair, because the loss of both FEN1 and either POLβ or XRCC1 had an additive or synergistic impact on SSBR rate. Intriguingly, we observed some residual decline in H_2_O_2_-induced SSBs in cells lacking both FEN1 and either XRCC1 or POLβ, presumably reflecting the activity of an alternative repair pathway.

Finally, it is worth noting that FEN1 is not universally required for the rapid rates of SSBR. Unlike MMS and H_2_O_2_, FEN1 deletion did not affect the rate of repair of CPT-induced SSBs resulting from abortive TOP1 activity during treatment. This may reflect that gap filling is not necessary at TOP1-induced SSBs, which consist of a nick at which TOP1 polypeptide is covalently linked to the 3′-terminus via a 3′-phosphotyrosyl bond.

In summary, our results reinforce the distinct roles of XRCC1–POLβ and FEN1–PCNA–POLD1 in short-patch and long-patch repair in human cells. However, these results demonstrate that rather than being a back-up pathway, FEN1-dependent long-patch repair is required for the rapid repair of SSBs following DNA alkylation and DNA oxidation, particularly in G1 phase of the cell cycle.

## Supplementary Material

gkaf710_Supplemental_File

## Data Availability

The data underlying this study are available in the article and its online supplementary material.
