# Supplementary material for: FEN1 is critical for rapid single-strand break repair in G1 phase
Source: Nucleic Acids Res. 2025 Jul 22;53(14):gkaf710. doi: 10.1093/nar/gkaf710 (PMC12282947; doi:10.1093/nar/gkaf710)
Supplement: gkaf710_Supplemental_File [file gkaf710_supplemental_file.pdf]

Supplementary Data

FEN1 is Critical for Rapid Single-Strand Break Repair in G1 Phase

Burdova et al.

Supplementary Table S1. Primary and secondary antibodies

Supplementary Figure S1. Related to Figure 1

Supplementary Figure S2. Related to Figure 2

Supplementary Figure S3. Related to Figure 3

Table S1. Primary and secondary antibodies

**Primary antibodies**	**Source**	**Identifier**
MAR/PAR	Cell Signaling	#83732
PCNA	Santa Cruz	sc-56
PCNA	Abcam	ab252848
MCM2	BD Biosciences	610700
FEN1	Life Technologies	MA1-23228
XRCC1	Novus Biologicals	NBP1-87154
Histone 3 (H3)	Abcam	ab10799
Polβ	Millipore	6C0087
Importin β	Santa Cruz	sc-137016
Histone 2B (H2B)	Abcam	ab1790
Histone 4 (H4)	Cell Signaling	#2935
PARP1	Santa Cruz	sc-8007
Biotin	Cell Signaling	#5597
**Secondary antibodies**	**Source**	**Identifier**
Goat anti-mouse HRP	Biorad	170-6516
Goat anti-rabbit HRP	Biorad	170-6516
Donkey anti-mouse AlexaFluor 647	Life Technologies	A31571
Donkey anti-rat AlexaFluor 568	Life Technologies	A78946
Donkey anti-rabbit AlexaFluor 488	Life Technologies	A21206

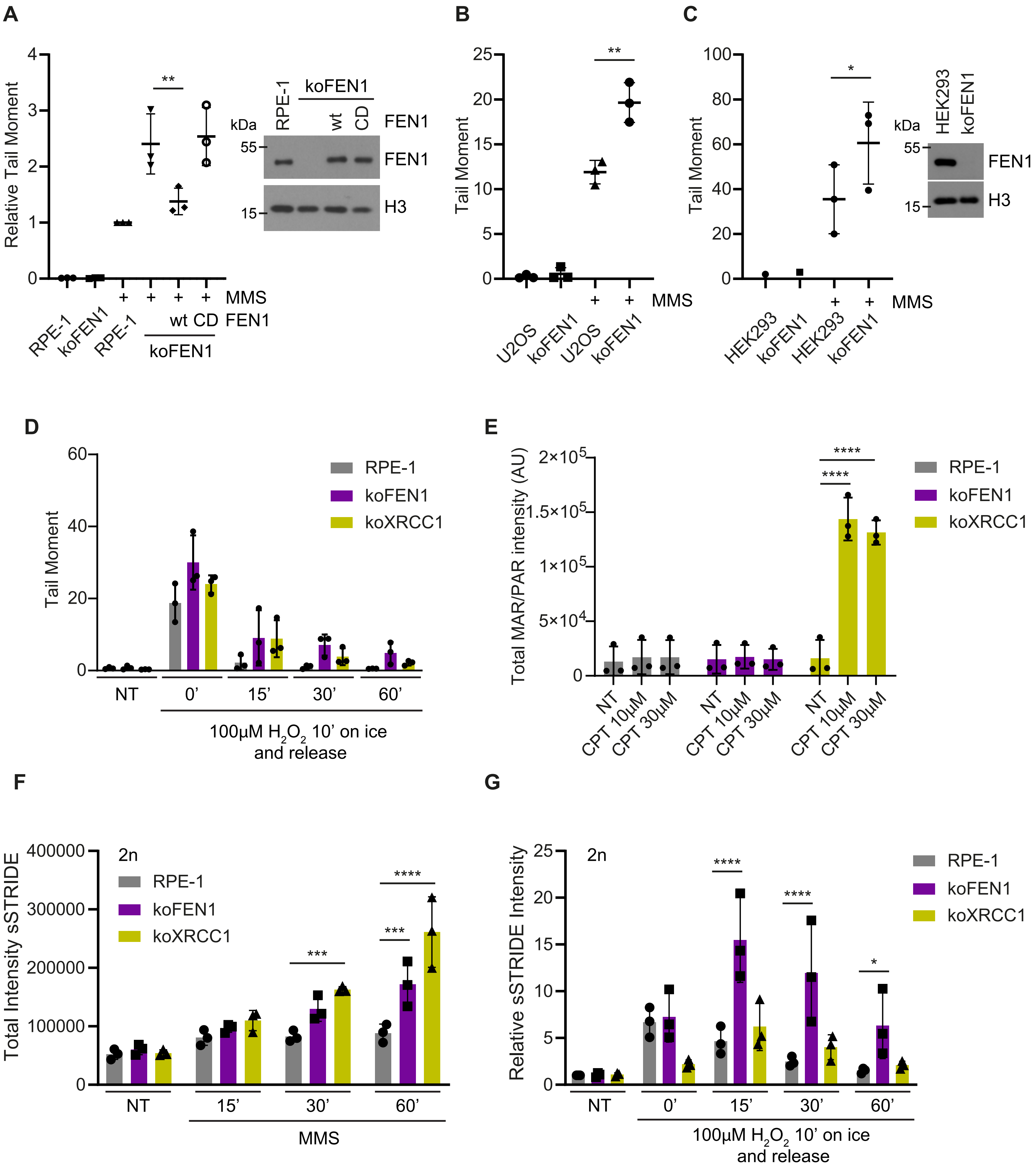
A
Relative Tail Moment
RPE-1
koFEN1
RPE-1
wt CD FEN1
koFEN1
MMS
**
kDa
55
15
FEN1
H3
B
Tail Moment
U2OS
koFEN1
U2OS
koFEN1
MMS
**
C
Tail Moment
HEK293
koFEN1
HEK293
koFEN1
MMS
*
kDa
55
15
FEN1
H3
D
Tail Moment
RPE-1
koFEN1
koXRCC1
NT
0'
15'
30'
60'
100µM H_2O_2 10' on ice and release
E
Total MAR/PAR intensity (AU)
2×10^5
1.5×10^5
1×10^5
5×10^4
RPE-1
koFEN1
koXRCC1
NT
CPT 10µM
CPT 30µM
****
****
F
Total Intensity sSTRIDE
2n
RPE-1
koFEN1
koXRCC1
NT
15'
30'
60'
MMS
***
***
****
G
Relative sSTRIDE Intensity
2n
RPE-1
koFEN1
koXRCC1
NT
0'
15'
30'
60'
100µM H_2O_2 10' on ice and release
****
****
*

Supplementary Figure S1. Related to Figure 1

(**A**) DNA strand breaks in asynchronous wild-type (RPE-1) cells, FEN1-deleted (koFEN1) RPE-1 cells, and FEN1-deleted cells complemented with either wild-type (wt) or catalytically dead (CD) FEN1 by proteofection, measured by alkaline comet assays. Cells were untreated (NT) or treated with 0.1 mg/ml methyl methanesulfonate (MMS) for 15 min at 37°C.
(**B**) DNA strand breaks in asynchronous wild-type (U2OS) and FEN1-deleted (koFEN1) U2OS cells, measured by alkaline comet assays in untreated (NT) and MMS-treated (0.1 mg/ml for 15 min at 37°C) conditions.
(**C**) DNA strand breaks in asynchronous wild-type (HEK293) and FEN1-deleted (koFEN1) HEK293 FlipIN Trex cells, measured by alkaline comet assays in untreated (NT) and MMS-treated (0.1 mg/ml for 15 min at 37°C) conditions.
(**D**) DNA strand breaks in asynchronous wild-type (RPE-1), FEN1-deleted (koFEN1), and XRCC1-deleted (koXRCC1) RPE-1 cells, measured by alkaline comet assays in untreated (NT) cells or after exposure to 100 μM hydrogen peroxide (H_2O_2) on ice for 10 min, followed by release into drug-free media at 37°C for the indicated times. Absolute tail moments corresponding to the data presented in Figure 1B are shown.
(**E**) Chromatin-associated ADP-ribosylation (MAR/PAR) detected by indirect immunofluorescence in asynchronous wild-type (RPE-1), FEN1-deleted (koFEN1), and XRCC1-deleted (koXRCC1) RPE-1 cells under untreated (NT) conditions or after treatment with 10 μM or 30 μM camptothecin (CPT) for 45 min at 37°C.
(**F**) Detection of single-strand breaks (SSBs) using sSTRIDE and indirect immunofluorescence in the 2n population of asynchronous wild-type (RPE-1), FEN1-deleted (koFEN1), and XRCC1-deleted (koXRCC1) RPE-1 cells under untreated (NT) conditions or following MMS treatment for the indicated times at 37°C.
(**G**) Detection of SSBs using sSTRIDE and indirect immunofluorescence in the 2n population of asynchronous wild-type (RPE-1), FEN1-deleted (koFEN1), and XRCC1-deleted (XRCC1) RPE-1 cells under untreated (NT) conditions or after treatment with 100 μM H_2O_2 in serum-free media on ice for 10 min, followed by release into fresh full media at 37°C for the indicated times.

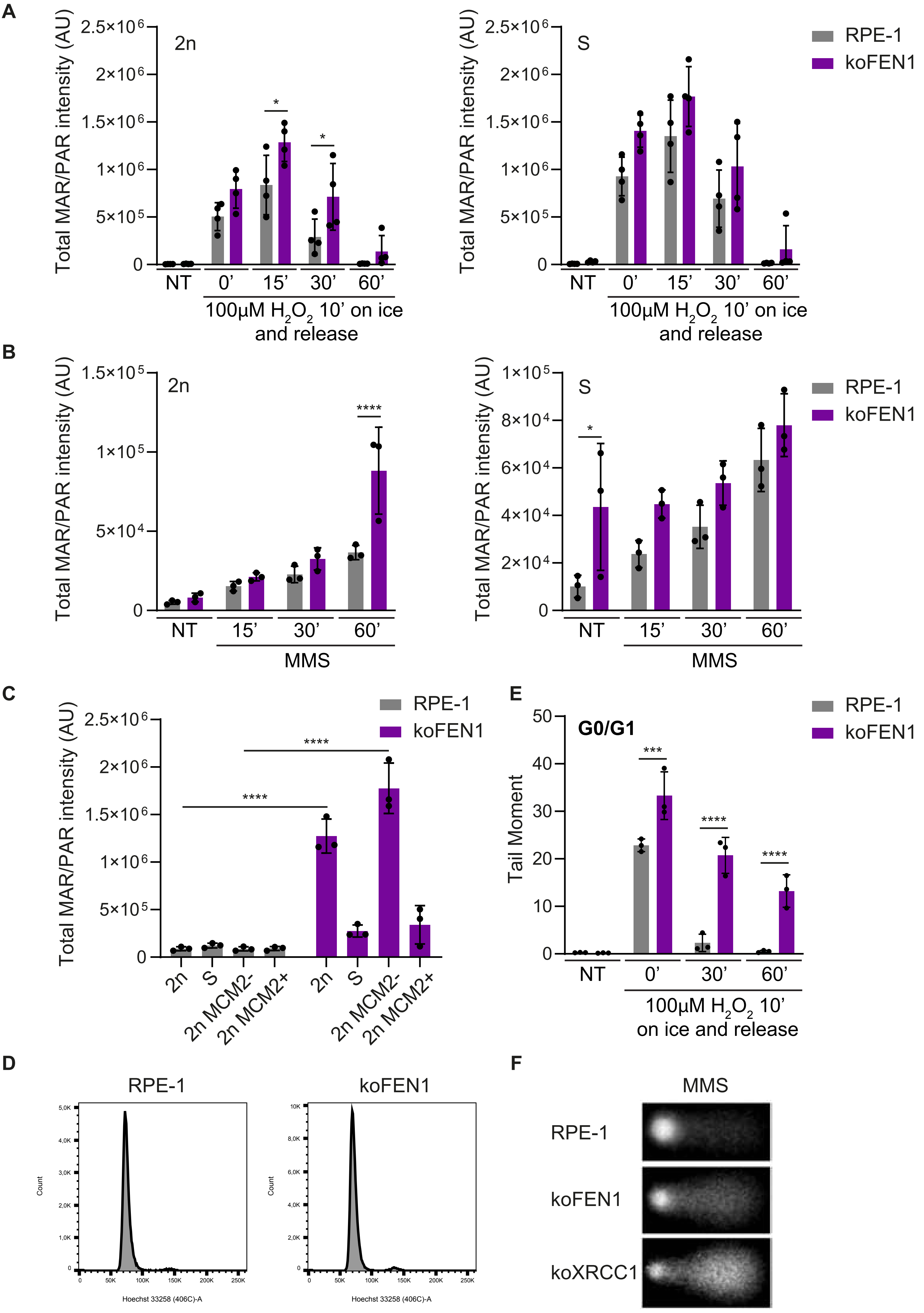
A
Total MAR/PAR intensity (AU)
2n
S
RPE-1
koFEN1
2.5×10⁶
2×10⁶
1.5×10⁶
1×10⁶
5×10⁵
0
*
NT
0'
15'
30'
60'
100µM H_2O_2 10' on ice and release
B
1.5×10⁵
1×10⁵
5×10⁴
8×10⁴
6×10⁴
4×10⁴
2×10⁴
****
MMS
C
2n
S
2n MCM2-
2n MCM2+
E
G0/G1
Tail Moment
50
40
30
20
10
***
100µM H_2O_2 10' on ice and release
D
RPE-1
koFEN1
Count
Hoechst 33258 (406C)-A
F
MMS
koXRCC1

Supplementary Figure S2. Related to Figure 2

(**A**) Quantification of chromatin-associated ADP-ribosylation (MAR/PAR) by indirect immunofluorescence in asynchronous cell populations. Wild-type (RPE-1) and FEN1-deleted (koFEN1) cells were untreated (NT) or treated with 100 μM hydrogen peroxide (H_2O_2) on ice for 10 min, followed by incubation in drug-free media at 37°C for the indicated times. Cell cycle phases were distinguished by DNA content (DAPI staining): G1 cells (2n) are shown in the left panel; S phase cells (S) are shown in the right panel.
(**B**) Quantification of chromatin-associated ADP-ribosylation (MAR/PAR) by indirect immunofluorescence in asynchronous cell populations. Wild-type (RPE-1) and FEN1-deleted (koFEN1) cells were untreated (NT) or treated with 0.1 mg/ml methyl methanesulfonate (MMS) for the indicated times. Cell cycle phases were distinguished by DNA content (DAPI staining): G1 cells (2n) are shown in the left panel; S phase cells (S) are shown in the right panel.
(**C**) Quantification of chromatin-associated ADP-ribosylation (MAR/PAR) by indirect immunofluorescence in asynchronous wild-type (RPE-1) and FEN1-deleted (koFEN1) cells treated with 100 μM H_2O_2 in serum-free media on ice for 10 min, followed by release into fresh full media at 37°C for 1 h. Cell cycle phases were distinguished by DNA content (DAPI staining) and MCM immunostaining, as indicated. Cells in G1 (2n; determined by DAPI staining), early G1 (2n MCM2-; determined by DAPI & MCM staining), late G1 (2n MCM2+; determined by DAPI & MCM staining), and S phase (S; determined by DAPI staining) are shown.
(**D**) FACS analysis of cell cycle profiles of G0/G1-synchronized wild-type (RPE-1) and FEN1-deleted (koFEN1) RPE-1 cells.
(**E**) DNA strand breaks in wild-type (RPE-1) and FEN1-deleted (koFEN1) RPE-1 cells in G1 phase, measured by alkaline comet assays. Cells were untreated (NT) or treated with 100 μM H_2O_2 on ice for 10 min, followed by release into drug-free media at 37°C for the indicated times. Absolute tail moments corresponding to the data presented in Figure 2G are shown.
(**F**) Representative comet assay images of in wild-type (RPE-1), FEN1-deleted (koFEN1), and XRCC1-deleted (koXRCC1) RPE-1 cells in G1 phase following treatment with 0.1 mg/ml MMS for 15 min at 37°C.

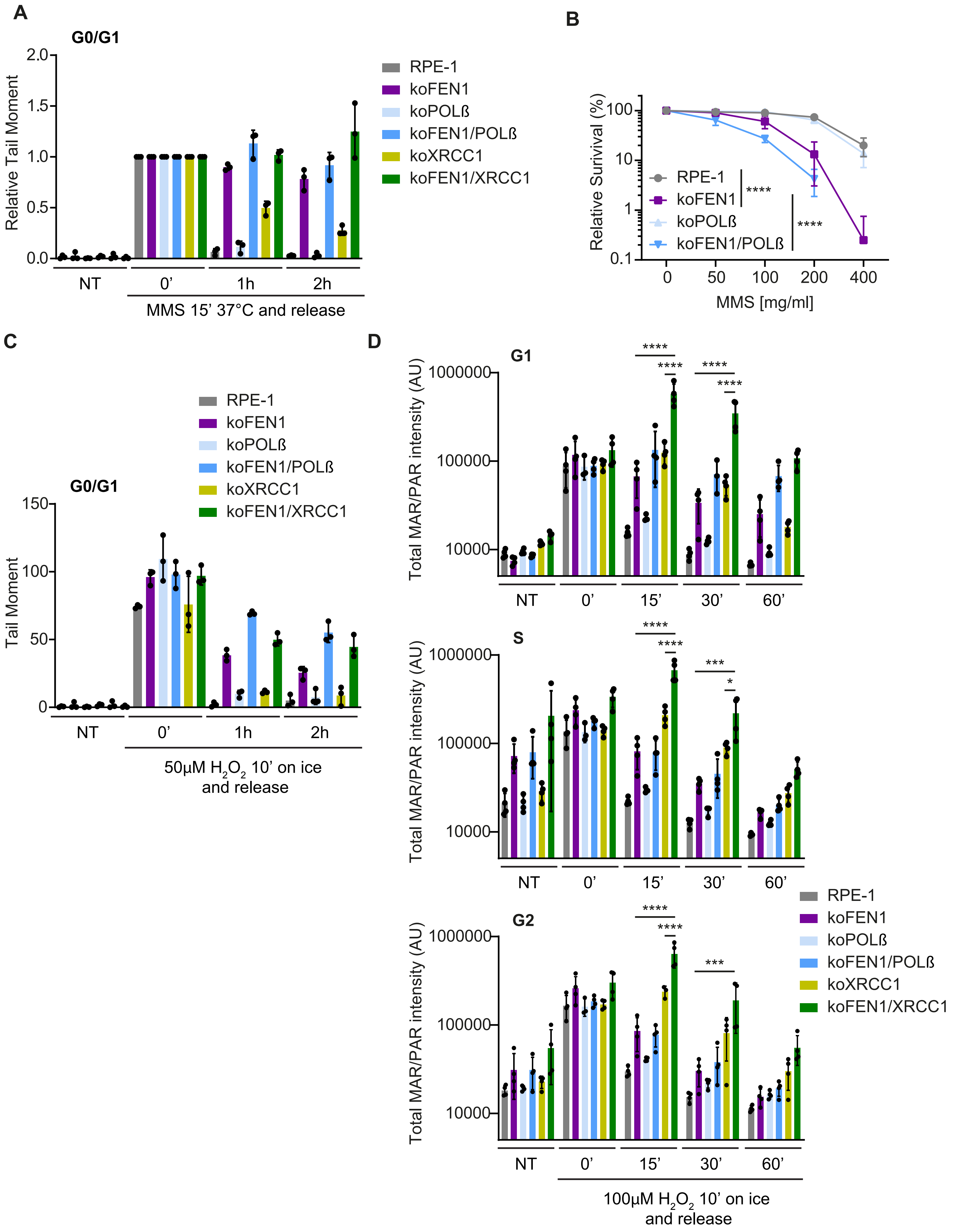

A
G0/G1
Relative Tail Moment
2.0
1.5
1.0
0.5
0.0
NT
0'
1h
2h
MMS 15' 37°C and release
RPE-1
koFEN1
koPOLß
koFEN1/POLß
koXRCC1
koFEN1/XRCC1
B
Relative Survival (%)
100
10
1
0.1
RPE-1
koFEN1
koPOLß
koFEN1/POLß
****
****
0
50
100
200
400
MMS [mg/ml]
C
G0/G1
Tail Moment
150
100
50
0
NT
0'
1h
2h
50µM H_2O_2 10' on ice and release
RPE-1
koFEN1
koPOLß
koFEN1/POLß
koXRCC1
koFEN1/XRCC1
D
G1
Total MAR/PAR intensity (AU)
1000000
100000
10000
NT
0'
15'
30'
60'
S
G2
100µM H_2O_2 10' on ice and release
RPE-1
koFEN1
koPOLß
koFEN1/POLß
koXRCC1
koFEN1/XRCC1

Supplementary Figure S3. Related to Figure 3

(**A**) DNA strand breaks in the indicated wild-type (RPE-1) and single/double knockout RPE-1 cells in G1 phase, measured by alkaline comet assays. Cells were untreated (NT) or treated with 0.1 mg/ml methyl methanesulfonate (MMS) for 15 min at 37°C, followed by incubation in drug-free media for the indicated times. Relative tail moments are shown (corresponding absolute tail moments are presented in Figure 3C).
(**B**) Clonogenic cell survival of the indicated wild-type (RPE-1) and single/double knockout RPE-1 cells synchronized in G0/G1 phase after treatment with MMS.
(**C**) DNA strand breaks in the indicated wild-type (RPE-1) and single/double knockout RPE-1 cells in G1 phase, measured by alkaline comet assays. Cells were untreated (NT) or treated with 50 μM hydrogen peroxide (H_2O_2) on ice for 10 min, followed by incubation in drug-free media at 37°C for the indicated times. Absolute tail moments are shown (relative tail moments are presented in Figure 3D).
(**D**) Quantification of chromatin-associated ADP-ribosylation (MAR/PAR) by indirect immunofluorescence in asynchronous cells, untreated (NT) or treated with 100 μM H_2O_2 on ice for 10 min, followed by incubation in drug-free media at 37°C for the indicated times. Cell cycle phases were distinguished by DNA content (DAPI staining) and MCM immunostaining, as indicated. Cells in G1 (2n MCM2-; determined by DAPI & MCM staining), S-phase (S; determined by DAPI staining) and G2 phase (4n; determined by DAPI staining) are shown.
